# Clinical Utility of ^18^Fluorodeoxyglucose Positron Emission Tomography/Computed Tomography (^18^F-FDG PET/CT) in Multivisceral Transplant Patients

**DOI:** 10.3390/jimaging9060114

**Published:** 2023-06-07

**Authors:** Shao Jin Ong, Lisa M. Sharkey, Kai En Low, Heok K. Cheow, Andrew J. Butler, John R. Buscombe

**Affiliations:** Addenbrookes Hospital, Cambridge Universities Hospitals NHS Foundation Trust, Cambridge CB2 0QQ, UK; lisa.sharkey@nhs.net (L.M.S.); heok.cheow@addenbrookes.nhs.uk (H.K.C.); andrew.butler13@nhs.net (A.J.B.); jrb.wjnm@googlemail.com (J.R.B.)

**Keywords:** ^18^F-FDG 18F-Fluorodeoxyglucose Positron Emission Tomography (^18^F FDG-PET/CT), multivisceral transplant (MVTx), rejection, sepsis, post-transplant lymphoproliferative disorder (PTLD), transplant

## Abstract

Multivisceral transplant (MVTx) refers to a composite graft from a cadaveric donor, which often includes the liver, the pancreaticoduodenal complex, and small intestine transplanted en bloc. It remains rare and is performed in specialist centres. Post-transplant complications are reported at a higher rate in multivisceral transplants because of the high levels of immunosuppression used to prevent rejection of the highly immunogenic intestine. In this study, we analyzed the clinical utility of 28 ^18^F-FDG PET/CT scans in 20 multivisceral transplant recipients in whom previous non-functional imaging was deemed clinically inconclusive. The results were compared with histopathological and clinical follow-up data. In our study, the accuracy of ^18^F-FDG PET/CT was determined as 66.7%, where a final diagnosis was confirmed clinically or via pathology. Of the 28 scans, 24 scans (85.7%) directly affected patient management, of which 9 were related to starting of new treatments and 6 resulted in an ongoing treatment or planned surgery being stopped. This study demonstrates that ^18^F-FDG PET/CT is a promising technique in identifying life-threatening pathologies in this complex group of patients. It would appear that ^18^F-FDG PET/CT has a good level of accuracy, including for those MVTx patients suffering from infection, post-transplant lymphoproliferative disease, and malignancy.

## 1. Introduction

Multivisceral transplant (MVTx) is the transplantation of both foregut and midgut in which the native abdominal viscera are resected from the recipient; then, a composite graft from a cadaveric donor, which often includes the liver, the pancreaticoduodenal complex and small intestine, is transplanted en bloc [1,2]. The stomach, spleen, colon and one or both kidneys may also be transplanted simultaneously. The procedure is still rare and is performed in specialist centers. The indications for this procedure include those patients with permanent intestinal failure [3] and its associated complications, including intestinal-failure-associated liver disease (IFALD), hepatic cirrhosis with porto-mesenteric venous thrombosis, making an isolated liver transplant technically impossible [4], and an acute occlusion of both the celiac axis and superior mesenteric artery. Since 2000, the use of MVTx has been increasing [5], primarily due to improvements in graft and recipient survival from better surgical techniques, and postoperative management, which often requires significant immunosuppression regimens.

In the early post-operative period, sepsis and acute rejection are the most common complications. Those patients that survive the acute post-operative period may suffer similar problems to other solid-organ transplant recipients, including chronic rejection, graft ischemia, post-transplant lymphoproliferative disorder (PTLD) and infection. The latter two have been reported at a higher rate in MVTx because of the high levels of immunosuppression that are required, as a significant amount of gut lymphoid tissue is transplanted along with the graft [6].

The diagnosis of these late post-operative complications relies on a variety of techniques, including examinations of blood and tissue from the patient along with imaging, which normally includes ultrasound and computed tomography (CT) [2].

Nuclear medicine techniques for imaging infection, even in transplanted patients, have relied on the use of labelled leucocytes [7], but this technique relies on competent leucocytes and has been found to be problematic in the immunosuppressed [8,9].

In the last decade, ^18^F Fluoro-de-oxy glucose positron emission tomography (^18^F-FDG PET) has demonstrated good accuracy in immunosuppressed patients, including iatrogenic immunosuppression, and has been used to identify post-transplant lymphoproliferatice disorder (PTLD) [10].

The aim of this study was to analyse and evaluate the utility of ^18^F-FDG PET/CT performed for clinical reasons in MVTx recipients in whom infection, PTLD or other conditions, such as rejection, could cause a fever of unknown cause requiring further investigation where other imaging modalities, such as CT and ultrasound, were unable to provide a reasonably confident answer.

## 2. Materials and Methods

### 2.1. Study Design

This was an open-label retrospective study of all patients with an MVTx in whom an ^18^F-FDG PET/CT was performed to identify or rule out a possible cause of post-transplant complications. These studies were performed after other investigations, including CT, had failed to answer the specific clinical question that had been set by their attending physicians. All studies complied with the British national guidelines for PET/CT and were considered routine clinical imaging examinations.

### 2.2. Patients

From December 2008 to June 2014, 20 multivisceral transplant patients were referred for a total of 28 ^18^F-FDG PET/CT examinations with 40 specific clinical queries (Table 1). Eighteen clinical queries were related to the identification of a source of sepsis; 10 clinical queries were to rule out a specific anatomical site of infection; 6 examinations were looking for or delineating the extent of post-transplant lymphoproliferative disorder; 3 were cases of suspected rejection; 3 were identifying malignancy/staging of malignancy.

### 2.3. Imaging

During the review, the patients were initially studied on a GE 690 PET/CT (GE Milwaulkee, Milwaukee, WI, USA) from December 2008, and from January 2010 on a GE 690 PET/CT with time of flight. Between 162 and 396 (mean 349, SEM 8.5), MBq (4.4–10.7 mCi) ^18^F-FDG was injected intravenously, and imaging was commenced at 90+/− 5 min post injection. Each patient was imaged for between 6 and 8 bed positions with 3 min per bed position. Glycaemic control was variable in this patient group due to complications affecting their transplant pancreatic function, but plasma glucose was between 68.4 and 210.6 (mean 108.6, SEM 5.75) mg/dL, with two patients being scanned with a plasma glucose greater than 145 mg/dL.

### 2.4. ^18^F-FDG PET/CT Interpretation

The initial clinical reports were reviewed. All studies were reported or reviewed by a specialist trained in the interpretation of PET/CT. All studies were reported with clinical information and other previous imaging available. All reports were available on the day of the investigation.

### 2.5. Clinical Assessment of Examination Results and Utility

The clinical notes from each patient were retrieved and reviewed at the end of the study period by the surgical, medical and radiology teams. Specific indications and thought processes leading to the request for each of these examinations were examined Based on the clinical notes, it was determined whether the final clinical diagnosis, obtained from the results of pathology, microbiology, biochemistry and response to specific therapies, correlated with the initial clinical query and the clinical report of the ^18^F-FDG PET/CT performed for the patient. The joint review was also utilized to determine if the result of the ^18^F-FDG PET/CT resulted in a change in clinical management, for example by the initiation of new treatments or the cessation of on-going treatments, as well as any change in further surgical management. This included the cancelation of planned surgery.

## 3. Results

### 3.1. General

A total of 28 studies were performed in 20 patients; 4 patients underwent 2 studies and 2 patients had 3 studies. A diagnosis was identified in 16 patients: 8 were identified as having an infection, 2 were identified as PTLD, 2 identified as rejection, 2 as malignancy and 2 with no abnormalities demonstrated. Final clinical diagnosis was established in 12 patients related to 16 scans. Where a final diagnosis can be confirmed, the accuracy of ^18^F-FDG PET/CT was determined as 66.7% (8/12). There were no false-negative and 2 false-positive (both patients were imaged for rejection) results. 

There were 24 scans that affected patient management, of which 9 related to starting new treatments or directing investigations that resulted in a change of treatment. There were also six cases in which the ^18^F-FDG PET/CT scan report resulted in an ongoing treatment or planned surgery being stopped.

### 3.2. Sepsis

Of the 18 examinations performed to identify the source of sepsis, the median age of the patients was 48 (range 28–62). They all had a prior relevant CT examination (which could not provide a reasonably confident clinical answer) conducted a median of 7 days (range 4–69) prior to the ^18^F-FDG PET/CT examinations. In 14 of the cases, the results of the ^18^F-FDG PET/CT changed the clinical management of the patient.

**Figure 1:**
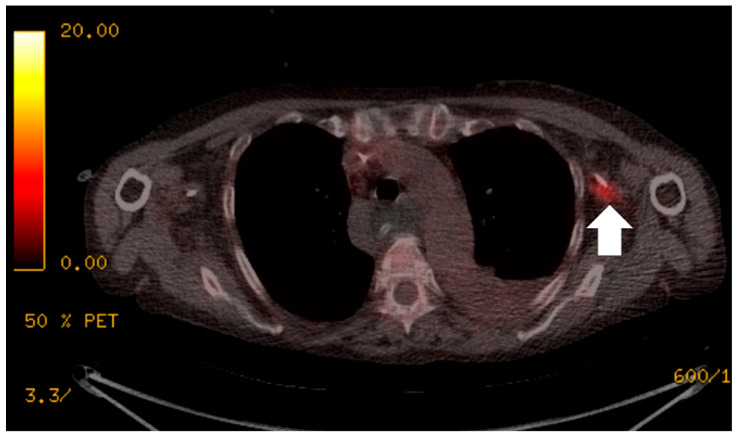
The patient presented with an elevated white cell count and raised C-reactive protein (CRP) of greater than 250 mg/L, but multiple previous image-guided drains, aspirations, line and blood cultures were negative. ^18^F-FDG PET/CT localised the infective focus to the left peripherally inserted central catheter (PICC) line (white arrow). The PICC line was removed and there was a subsequent improvement in the patient’s clinical condition.

**Figure 2:**
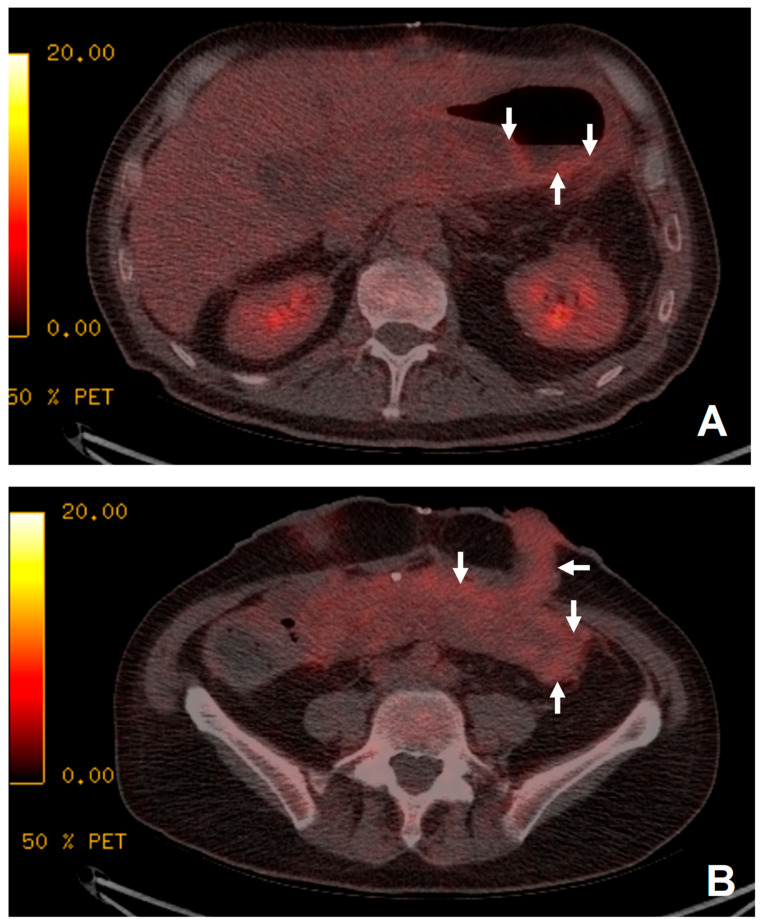
The patient presented with 1 week of pyrexia but no source of infection was identified. Increased uptake was demonstrated in the transplanted stomach (white arrows, image (**A**)) and small bowel (white arrows, image (**B**)) suggestive of rejection. Biopsy just prior to ^18^F-FDG PET/CT demonstrated a non-specific increase in apoptotic debris within the lamina propria of uncertain significance. As no focus of infection was demonstrated and the appearance of the transplanted stomach (**A**) and small bowel (**B**) was suggestive of rejection, the patient was treated for rejection with immune suppression. Following anti-rejection treatment, the patient’s clinical picture improved with resolution of the pyrexia. Subsequent biopsies taken 6 weeks later demonstrated no remaining features of rejection.

There were 17 examinations that could answer the clinical question regarding the possibility and the source of sepsis. Among these, seven of the examinations localized the source of sepsis (Figure 1). Of these, 10 examinations were able to exclude the presence of a source of sepsis to account for the patient’s symptoms, but incidental findings of rejection were noted in two patients (Figure 2) and myositis in one patient. The remaining examination was deemed unhelpful by the referring clinical team.

### 3.3. Specific Sites for Infection

Within this group, 10 examinations were performed to identify the specific anatomical sites for suspected foci of infection. The median age of these patients was 54 (range 46–62), and they had a relevant prior CT conducted a median of 6 days (range 4–136) prior to the ^18^F-FDG PET/CT examination. All 10 examinations were deemed by the clinical team to have answered the clinical query.

In eight of the cases, the results of the ^18^F-FDG PET/CT changed the clinical management plan of the patient.

**Figure 3:**
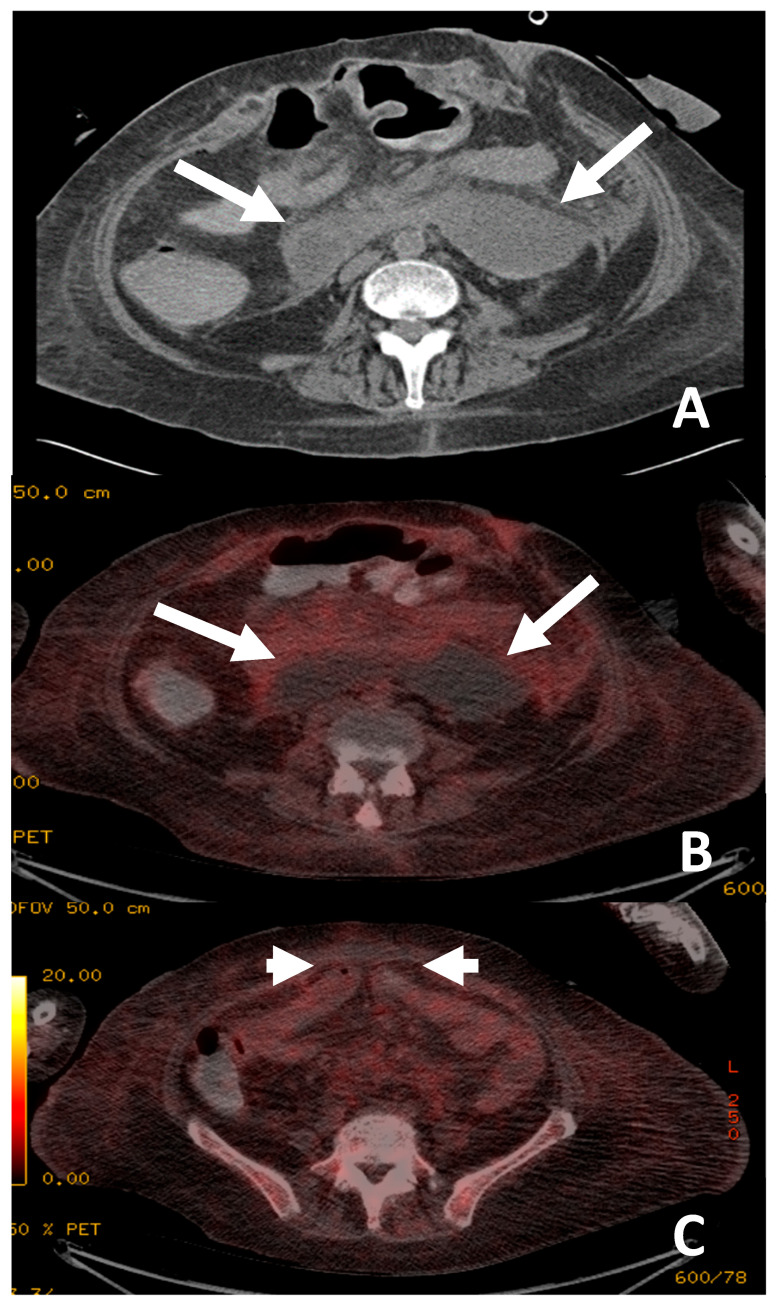
The patient presented with continued pyrexia and a white cell count of 40 × 10^9^cells/L. CT from 4 days prior demonstrated a lesser sac collection (**A**) with clinical concerns that the lesser sac collection (white arrows, image (**A**)) may be the source of sepsis and the permacol gusset mesh used for closure may be infected. ^18^F-FDG PET/CT (**B**,**C**) demonstrated the collection in the lesser sac (white arrows, image (**B**)) is likely to be innocuous as it did not show high tracer uptake and there was no significant uptake at the site of the permacol gusset closure material (short arrows, image (**C**)) to suggest an infected implant. The patient avoided a repeat operation for a surgical washout of the lesser sac collection and the removal of the closure mesh.

There were also five examinations that excluded infection in the specific anatomical compartments in question. One examination was performed to exclude infection in a lesser sac collection seen on a CT four days prior as well as infection in the surgical mesh used for abdominal closure (Figure 3). The lesser sac collection and the surgical graft demonstrated low ^18^F-FDG uptake, excluding infection at these sites; thus, the patient avoided surgical washout and removal of the gusset. Another patient’s examination was performed to exclude infected right flank collection as well as a coeliac graft. As no uptake was demonstrated in either the collection or the graft, the patient’s surgical washout and graft refashioning was cancelled. In a third examination patient who had a CT-identified fluid collection in the right flank that was not ^18^F-FDG avid, the patient also avoided a surgical washout. The fourth patient was seen on CT to have a suspected infected thrombus along the left peripherally inserted central catheter (PICC) line (Figure 1). The ^18^F-FDG PET/CT was performed on a subsequent febrile episode two months later, and showed no ^18^F-FDG avid thrombus, although in this case management was not affected. In the fifth patient, a negative ^18^F-FDG PET/CT demonstrated that a previously seen pelvic collection was also not infected. The patient avoided a change in the peritoneal dialysis line.

In one patient with suspected sepsis, rejection was suggested on the ^18^F-FDG PET/CT and this was confirmed on biopsy (Figure 2).

### 3.4. Post-Transplant Lymphoproliferative Disorder

A total of six examinations were performed on four patients with the median age of 26 (range 23–56) to delineate the extent of PTLD. All the examinations could answer the clinical question and affected the management of the patients.

**Figure 4:**
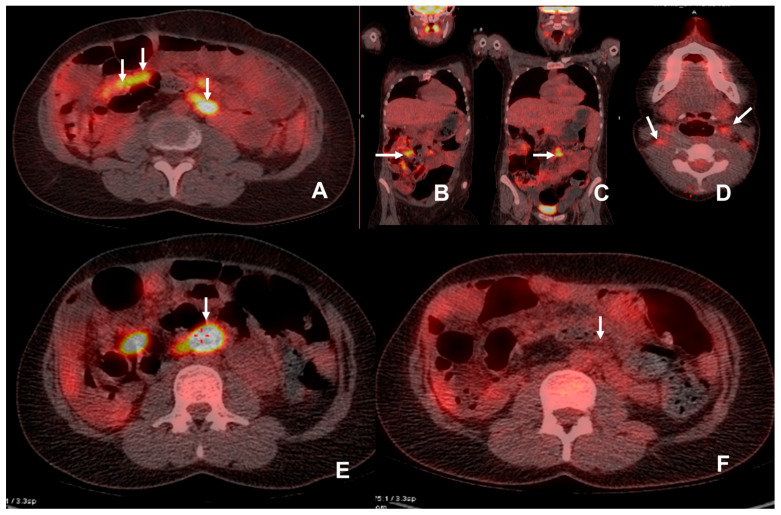
The patient was admitted with an elevated amylase level 6 years post multivisceral transplant. CT demonstrated increased prominence of mesenteric lymph nodes and there was a clinical suspicion of PTLD. ^18^F-FDG PET/CT demonstrated multiple FDG avid (SUVmax 12.6) mesenteric lymph nodes (white arrows)(**A**–**C**). Biopsy demonstrated a monomorphic plasma cell infiltration in keeping with PTLD. Anti-rejection/immunosuppressant therapy was reduced in an attempt to control the PTLD. Follow-up ^18^F-FDG PET/CT examination was performed at 2.5 months (**D**,**E**) due to an inability to biopsy the deep-seated lymph node in a difficult abdomen with significant thrombocytopenia. This showed a marked increase in SUV (SUVmax 23.7) and the size of the mesenteric lymph nodes, a large FDG avid para-aortic lymph node (white arrow with adjacent red crosshairs, image (**E**)) and additional involvement of cervical lymph nodes (white arrows, image (**D**)) with SUVmax at 4.6, in keeping with progressive PTLD. Results of this examination were discussed at MDT and Rituximab was commenced. A further follow-up ^18^F-FDG PET/CT was performed 10 weeks after the initiation of Rituxiamb and demonstrated resolution with a significant drop in SUV (**F**) for the reference nodes.

The first patient was initially examined by CT for increased amylase levels 6 years post transplant and subsequently interrogated with ^18^F-FDG PET/CT, which showed small bowel and mesenteric lymph node uptake (Figure 4). This was followed by biopsy of the small bowel mucosa which demonstrated a monoclonal plasma cell infiltration in keeping with PTLD. The immunosuppressant therapy was reduced in an attempt to halt the progression of PTLD. One month later, the patient re-presented with neck lymphadenopathy with suspicious appearances on ultrasound. This lymphadenopathy was ^18^F-FDG avid, and the patient was started on Rituximab. A third ^18^F-FDG PET/CT scan demonstrated a good response.

Three remaining patients had findings of enlarged intra-abdominal lymph nodes and bowel-wall-thickening on CT suggestive of PTLD. In two of these cases, the clinical suspicion of PTLD was heightened by a recent increase in Epstein Barr virus (EBV) titres. All three patients underwent ^18^F-FDG-PET/CT examinations, which were negative for PTLD. Biopsy confirmed the absence of PTLD in two patients and in the third patient the negative ^18^F-FDG-PET/CT meant biopsy was avoided, as it would have been clinically challenging due to the significant platelet function derangement.

### 3.5. Rejection

**Figure 5:**
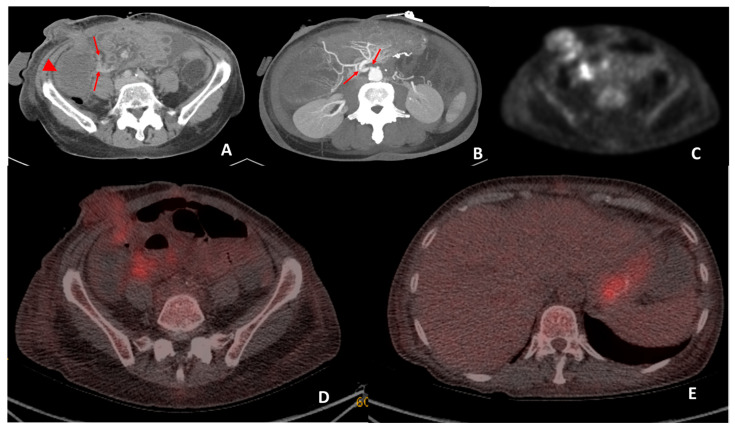
CT performed on day 19 post transplant for sepsis and query ischemia or rejection. Portal venous-phase CT imaging was performed on day 19 post transplant (**A**) demonstrating subtle mural oedema in the caecum (arrowhead) and sub centimeter mesenteric lymph nodes (arrows) surrounding the caecum. Maximum intensity projection (**B**) of the superior mesenteric origin on post-operative day 19 CT demonstrates a double kink (arrows) at this site. The patient subsequently underwent angiography and pressure measurements, which demonstrated a 27 mmHg pressure gradient between the aorta and SMA. Angioplasty was performed and reduced the pressure gradient to 21 mmHg. Attenuation-corrected ^18^F-FDG PET (**C**) demonstrated increased uptake in the mucosa of the small bowel surrounding the stoma and the mesenteric lymph nodes. Hybrid imaging ^18^F-FDG PET-CT demonstrated co-localisation of the increased uptake to the small bowel surrounding the stoma and also at the mesenteric lymph nodes (**D**). Biopsies performed on the small bowel surrounding the stoma were reported to be in keeping with mild rejection. The increased low-grade uptake in the stomach (**E**) was interpreted as physiological uptake.

There were three patients for whom a single examination each was performed for suspected rejection with a median age of 61 years (range 51–62). All three of the examinations answered the clinical question regarding rejection. In two patients, the negative ^18^F-FDG-PET/CTs was confirmed by subsequent biopsy. In the third patient (Figure 5), there was thought to be a clinical question of sepsis or rejection. The ^18^F-FDG-PET/CT demonstrated increased uptake in the mucosa of the small bowel surrounding the stoma and the mesenteric lymph nodes, which was interpreted as in keeping with the possible combination of infection, ischemia and rejection. A biopsy of the ^18^F-FDG avid areas showed rejection in the small bowel and ischemia in the colon.

### 3.6. Malignancy

There were three patients with a median age of 48 years, (range 47–62) imaged with a specific clinical query regarding the presence or spread of a co-existent malignancy, of which two studies were helpful.

The unhelpful study showed, in one patient, multiple liver metastases from mixed gastric leiomyosarcoma, duodenal adenocarcinoma and pancreatic neuroendocrine tumour. The cause of the multiple ^18^F-FDG avid lesions within the liver could not be determined or confirmed as a septic focus or metastases. The patient declined further investigation and died 8 weeks later.

The ^18^F-FDG-PET/CT was helpful in the pre-surgical staging of the second patient who presented with a signet ring carcinoma. The ^18^F-FDG-PET/CT was performed on the third patient, who presented with one week of pyrexia, a large left-sided pleural effusion, and elevated inflammatory markers. Multiple negative previous investigations confirmed the patient did not have metastatic disease. This patient was suspected to be suffering a co-existent malignancy, but the ^18^F-FDG-PET/CT scan suggested only gastric and small bowel rejection. The patient responded to anti-rejection therapy.

## 4. Discussion

This study shows that, despite the complexity of these patients, ^18^F-FDG-PET/CT can deliver an accuracy of 66.7% in identifying the cause of significant disease when there are targeted, specific clinical queries, and this can translate into a change in management in 86% of cases. This compares favorably with the other reported uses of ^18^F-FDG in other complex groups of immunosuppressed patients [11,12]. The reason for these complexities in MVTx patients is due to both the extent of the surgery involved and the need for significant immunosuppression. This immunosuppression involves a difficult balance between the high risk of rejection and the risk of infection or the development of malignancies such as PTLD. These patients often have multiple intra-venous lines, feeding lines and intra-abdominal drains, and, in the acute phase, all may be sites of sepsis. Often, these patients are also significantly cachectic with difficult access, rendering the indwelling lines, tubes and drains much more precious, as access may be lost once these are removed. The clinical symptoms may not be helpful, as patients present with similar symptoms to those found in infection and rejection. Anatomical imaging with CT can be particularly difficult, as these patients have suffered from chronic malnutrition so there are few fat planes, making it difficult to read intra-abdominal images [2]. Often, these patients have poor renal function, and intravenous contrast administration are omitted to preserve residual renal function, resulting in an even higher complexity in CT interpretation. Intra-abdominal and pelvic collections are often seen in this patient group and may be related to chyle leaks rather than infected collections; however, determining if any of these collections are infected can be more problematic.

^18^F-FDG-PET/CT could change management in 86% of our patients, which is vital as infection, especially in the weeks following transplantation, can be a major cause of mortality in these patients [13]. There is a diverse range of causes, for example, catheter-related, respiratory, wound and abdominal infection [13]. Paradoxically, acute cellular rejection leads to a loss of mucosal integrity and bacterial translocation, which may manifest as a systemic inflammatory response or sepsis. The investigative approach remains a painfully difficult subject. Changes in inflammatory markers are non-specific and can be a pathological marker or a post-surgical response. The grossly altered visceral and vascular transplant anatomy, the plethora of drains, tubes, lines and stents, and the significant amount of ascites, oedema and inflammatory fatty stranding remains a minefield for the reporting radiologist.

Although ^18^F-FDG is not an infection-specific tracer, it presents several advantages compared to cell-labelled imaging techniques [14]. The detection of infection by the labelled-leukocyte method depends on the migration of labelled neutrophils to the sites of infection, whereas ^18^F-FDG PET can demonstrate focal areas of increased metabolic activity without the need for leukocyte activation and migration. Labelled-leukocyte imaging is only effective when the predominant cellular reaction to infection involves neutrophils rather than lymphocytes and cell-labelling techniques depend on the patient having sufficient white cells and may not be feasible in neutropenic patients. ^18^F-FDG uptake is not dependent on such cellular migration and so should be considered the optimal choice for severely immunosuppressed patients. In our current study, where the clinical question was specific to the potential/putative site of infection based on the preceding CT examination, 10 out of 10 of the ^18^F-FDG PET/CTs that were performed were able to definitively confirm the site of infection or exclude focal infection at the site of concern. This supports the strong clinical utility of utilizing ^18^F-FDG PET/CT for imaging in immunosuppressed MVTx patients when there are specific focal concerns.

PTLD is usually caused by a proliferation of recipient B-lymphocytes, often driven by EBV. It may be nodal or extra-nodal, limited to allograft, limited to another organ, or widely disseminated [15]. The early identification of PTLD and staging is vital and it can be treated by a step-wise reduction in immunosuppression based on the extent of the disease in conjunction with the use of anti-CD20 monoclonal antibodies (Rituximab) [16,17].

PTLD itself is a histopathologically heterogeneous disease and ranges from benign polyclonal and polymorphic B-cell proliferation to malignant monoclonal lymphomatous lesions, which can be polymorphic or monomorphic. It has been reported that some lymphomas with very low or negative ^18^F-FDG uptake can lead to underestimation of the extent of the disease [18,19]. The role ^18^F-FDG PET/CT remains controversial, with mixed results being reported. One series found that ^18^F-FDG PET/CT demonstrated absolutely no uptake in mucosal-associated lymphoid tissue (MALT) lymphoma [18]. In a small series of four patients, ^18^F-FDG PET/CT was used to assess the metabolic response in PTLD following liver transplantation; however, this study only focused on monomorphic PTLD [17]. Another series assessed the role of ^18^F-FDG PET/CT in the staging and treatment assessment of monomorphic and polymorphic PTLD [10].

Other issues reported with ^18^F-FDG PET/CT imaging in PTLD include the presence of diffuse pulmonary involvement, non-typical organ invasion and concomitant infectious problems, which are atypical for lymphomatous proliferation in the immunocompetent patient and may be due to the immunosuppression regime. This means that, at present, ^18^F-FDG PET/CT is not recommended in recent guidelines for imaging PTLD [16]. However, a more recent study of 170 post-transplant ^18^F-FDG PET/CT scans demonstrates a sensitivity and specificity of 90% and 89%, respectively, for PTLD [20].

Although only six ^18^F-FDG PET/CT studies were performed for PTLD in our group, partly related to the application of guidelines that recommend contrast-enhanced CT, we were able to rule out PTLD in three patients and successfully used it to monitor treatment response in the only ^18^F-FDG PET/CT-positive patient who was found to have PTLD.

Acute rejection and, to a lesser degree, chronic rejection are among the most injurious and common complications of MVTx. The small intestine is one of the most immunogenic organs to undergo transplantation and is one of a few transplanted organs that are non-sterile and exposed to the external environment. The intestine is considered heavily dependent on the innate immune system for defense from constant exposure to microbes [21].

In current clinical practice, both broad categories of rejection are principally diagnosed by histopathological changes in the allograft; acute rejection is usually diagnosed histologically with endoscopic biopsies that are routinely procured via ileostomy post transplantation. Chronic rejection is difficult to diagnose and may not be apparent without full thickness biopsies of the small bowel or at explant [22]. In the case of MVTx, additional biopsies, i.e., of the other graft organs, may be necessary.

The role of ^18^F-FDG PET/CT in diagnosing rejection remains unproven. It is difficult to utilize ^18^F-FDG in cardiac, liver and kidneys’ transplant rejection due to the variable physiological uptake of FDG in these organs. A recent study has reported on the utility of ^18^F-FDG PET/CT in 122 patients with unresolved symptoms in solid-organ transplant, which also demonstrated sensitivity, specificity, and positive and negative predictive values for ^18^F-FDG PET/CT in diagnosing malignancy vs. infection of 97, 84, 87, and 96%, respectively [23]. However, the overall numbers of published studies evaluating infection vs. malignancy in solid organ transplantation is still very small and generally focused on a limited number of patients [24].

The stomach and small bowel have little physiological uptake of FDG. While there are a few studies on the utility of ^18^F-FDG or ^18^F-DOPA PET/CT in small bowel pathology, these mainly focus on a pre-operative workup of small bowel neuroendocrine tumours [25,26] Therefore, in those patients with MVTx, ^18^F-FDG may have some utility, although it is non-specific and its uptake alone would not necessarily differentiate rejection from other pathology. In the two patients with small bowel rejection, ^18^F-FDG uptake was seen. It was also useful in excluding rejection in two patients thought to have rejection but found not to have this problem. To the best of our knowledge, due to the relative rarity of multivisceral transplant, there have been no other studies looking at the utility of using ^18^F-FDG PET/CT in this subgroup of patients when facing a diagnostic conundrum. Clearly, more data are needed, which could hopefully be obtained from prospective trials.

## 5. Conclusions

Though this is a single-site retrospective review of a non-controlled group of patients, this study shows that ^18^F-FDG PET/CT is a promising technique in identifying life-threatening pathologies in this complex and often very sick group of patients. It would appear that ^18^F-FDG PET/CT has a good level of accuracy, including in those MVTx patients suffering from infection, PTLD, and malignancy. The role in rejection of stomach and small bowel transplant remains unproven, and further research in this area would be welcome.

## Figures and Tables

**Table 1:** Demographics of multivisceral transplant patients in the study.

Age (at Examination)		Average 45.7 Years; Range, 22.9–61.9 Years
Sex	Male	10
	Female	10
Indication for Multivisceral transplant	Primary cause	Numbers
	Thromboembolic disease	10
	Complication of Crohn’s disease	3
	Intra-abdominal Malignancy	1 (post Whipple’s procedure) 1 (small bowel resected with desmoid tumour) 1 (post-radiotherapy strictures)
	Visceral myopathy	2
	Volvolus	1
	Intussusception	1
	Small bowel obstruction secondary to adhesions	1

## Data Availability

Data not freely available due to restrictions from UK GDPR.
